# Exercise‐Induced Rhabdomyolysis With Markedly Elevated Creatine Kinase and Preserved Renal Function

**DOI:** 10.1155/crin/3900923

**Published:** 2026-07-01

**Authors:** Navuddh Oam, Sophareine Samphon, Pisey Say, Samith Sourn

**Affiliations:** ^1^ Medical Department, Vissar Medical and Nursing Center, Phnom Penh, Cambodia

**Keywords:** acute kidney injury, creatine kinase, exercise-induced rhabdomyolysis, preserved renal function, rhabdomyolysis

## Abstract

**Background:**

Exercise‐induced rhabdomyolysis results from skeletal muscle injury after strenuous or unaccustomed exertion. Acute kidney injury is a feared complication, but creatine kinase magnitude alone is an imperfect predictor of kidney injury.

**Case Presentation:**

A 24‐year‐old previously healthy male presented with dark urine after one week of high‐intensity resistance and eccentric exercise as a novice participant in structured high‐intensity training. He reported daily oral hydration of more than 2.5 L and denied creatine monohydrate use. Initial tests showed creatine kinase of 79,038 U/L, creatinine of 1.3 mg/dL, estimated glomerular filtration rate of approximately 79 mL/min/1.73 m^2^ by the 2021 CKD‐EPI creatinine equation, aspartate aminotransferase (AST) of 507 U/L, and urine dipstick blood of 3+ with few red blood cells. Urea, potassium, calcium, magnesium, and phosphorus were within reference ranges, and urinalysis showed only trace protein without leukocytes or nitrites. He received isotonic intravenous fluids without bicarbonate, diuretics, or renal replacement therapy. Creatinine decreased to 0.6 mg/dL within 24 h and remained stable; urine dipstick blood became negative by Day 2; creatine kinase declined to 722 U/L by Day 10 and 287 U/L by Day 16, while creatinine remained normal at 0.7 mg/dL and aminotransferases improved to AST of 25 U/L and ALT of 42 U/L.

**Conclusion:**

This case is best interpreted as severe exertional rhabdomyolysis with pigmenturia and preserved renal function, rather than confirmed intrinsic acute kidney injury. The report highlights the need to avoid creatine kinase–centric risk assessment and to interpret a mildly elevated admission creatinine in context, especially when rapid normalization follows fluid resuscitation.

## 1. Introduction

Rhabdomyolysis refers to skeletal muscle breakdown with release of intracellular constituents, including creatine kinase (CK), myoglobin, electrolytes, and aminotransferases, into the circulation. Myoglobin‐associated kidney injury may occur through renal vasoconstriction, oxidative tubular injury, and intratubular cast formation, particularly in the presence of hypovolemia and aciduria [1–3].

Exertional rhabdomyolysis occurs after strenuous, unaccustomed, prolonged, or eccentric exercise. The condition is increasingly recognized in young adults who participate in high‐intensity training programs. Although severe CK elevation often prompts concern for acute kidney injury (AKI), CK concentration by itself has limited ability to predict clinically important renal outcomes; risk assessment should include creatinine kinetics, urine output, electrolytes, acid–base status, volume status, and coexisting risk factors [4, 5].

The original purpose of this case is therefore not to present a novel mechanism of disease but to clarify a common clinical interpretation problem: a very high CK value with dark urine may lead clinicians to overdiagnose AKI when renal function is actually preserved. We report a young man with CK of 79,038 U/L and pigmenturia after intensive exercise, whose mildly elevated admission creatinine rapidly normalized after hydration. The case provides a practical teaching example on distinguishing severe exertional rhabdomyolysis from confirmed intrinsic kidney injury and on avoiding overinterpretation of a single creatinine value.

## 2. Case Presentation

A 24‐year‐old previously healthy male presented on 20 February 2026 to the emergency service of Vissar Medical and Nursing Center in Phnom Penh, Cambodia, because of dark‐brown urine first noticed on the day of admission. He reported that he was new to structured high‐intensity gym workouts and had completed approximately 1 week of resistance and eccentric training exercises. The training occurred in a tropical climate. He reported daily oral hydration of more than 2.5 L and did not describe deliberate fluid restriction; nevertheless, relative exercise‐associated fluid deficit from prolonged sweating and increased insensible loss remained clinically possible.

He denied recent trauma, febrile illness, alcohol consumption, illicit drug use, anabolic steroid use, and creatine monohydrate supplementation. He reported taking one scoop of whey protein supplementation daily. No other medication use was documented. There was no known family history of neuromuscular disease.

On arrival, he was alert and fully oriented. Vital signs were within normal limits. Physical examination revealed mild generalized muscle tenderness without focal weakness. There were no signs of compartment syndrome, including no tense muscle compartments, no neurovascular compromise, and no pain disproportionate to examination. Electrocardiography was normal, and chest radiography showed no acute abnormality.

Admission blood and urine samples supported severe exertional rhabdomyolysis with pigmenturia. The CK level was 79,038 U/L. Serum creatinine was 1.3 mg/dL, corresponding to an estimated glomerular filtration rate (eGFR) of approximately 79 mL/min/1.73 m^2^ using the 2021 CKD‐EPI creatinine equation. Because baseline creatinine was unavailable, formal confirmation of AKI based on change from baseline was not possible; however, the recorded body weight and height provided anthropometric context for renal interpretation. Urea and electrolytes were within reference ranges, including potassium, calcium, magnesium, and phosphorus. Urine dipstick was positive for blood (3+) with few red blood cells on microscopy, consistent with myoglobinuria rather than true hematuria.

The patient was admitted for supportive treatment. Isotonic normal saline was initiated promptly, targeting a urine output of 200–300 mL/hour. Bicarbonate alkalinization and diuretics were not used. Within the first 24 h, urine color improved from dark brown to light yellow. Serum creatinine decreased to 0.6 mg/dL by Day 2 and remained stable thereafter. Electrolyte levels remained stable throughout hospitalization. No hyperkalemia, metabolic acidosis, arrhythmia, oliguria, renal replacement therapy requirement, or compartment syndrome occurred.

The patient was discharged in stable condition with advice on oral hydration, avoidance of immediate high‐intensity exercise, gradual return to training, and follow‐up testing. Outpatient follow‐up laboratory testing on Day 16 showed continued biochemical recovery, with CK of 287 U/L, creatinine of 0.7 mg/dL, aspartate aminotransferase (AST) of 25 U/L, and alanine aminotransferase (ALT) of 42 U/L. Written informed consent for publication was obtained, and all data were anonymized.

## 3. Results

Admission laboratory evaluation demonstrated leukocytosis (WBC: 15.4 × 10^9^/L) with neutrophil predominance (78%) and relative lymphopenia (14%), consistent with a physiological stress response. Hemoglobin, hematocrit, red blood cell count, and platelet count were within normal limits (Table 1).

**TABLE 1:** Comprehensive admission laboratory results.

Parameter	Result	Reference range	Interpretation
*Hematology (CBC)*			
WBC (× 10^9^/L)	15.4	3.4–9.6	Leukocytosis
RBC (× 10^12^/L)	5.21	4.35–5.65	Normal
Hemoglobin (g/dL)	15.3	13.5–17.5	Normal
Hematocrit (%)	45	38–48	Normal
Platelets (× 10^9^/L)	240	157–371	Normal
Neutrophils (%)	78	44–71	Elevated
Lymphocytes (%)	14	17–42	Reduced

*Clinical chemistry*			
Creatine kinase (U/L)	79,038	39–308	Markedly elevated
Creatinine (mg/dL)	1.3	0.7–1.2	Mild elevation
Approximate eGFRcr (mL/min/1.73 m^2^)	79	Age/sex dependent	Not diagnostic of AKI alone
Urea (mmol/L)	5.7	2.1–7.1	Normal
Sodium (mmol/L)	141	135–145	Normal
Potassium (mmol/L)	4.2	3.5–5.1	Normal
Chloride (mg/dL)	103	98–107	Normal
Calcium (mg/dL)	9.4	8.8–10.2	Normal
Magnesium (mg/dL)	2.40	1.60–2.60	Normal
Phosphorus (mg/dL)	3.8	2.5–4.5	Normal
LDH (U/L)	1963	< 250	Markedly elevated

*Urine dipstick*			
Color	Dark brown	Yellow	Abnormal
Specific gravity	1.020	1.005–1.030	Normal
pH	6.0	4.5–8.0	Normal
Blood (dipstick)	3+	Negative	Positive
Protein	Trace	Negative	Mild
Leukocytes	Negative	Negative	Normal
Nitrites	Negative	Negative	Normal
Glucose	Negative	Negative	Normal
Ketones	Negative	Negative	Normal

*Note:* eGFRcr, estimated glomerular filtration rate based on creatinine; LDH, lactate dehydrogenase. eGFRcr was calculated using the 2021 CKD‐EPI creatinine equation and should be interpreted cautiously because serum creatinine may be non‐steady‐state during acute muscle injury.

Abbreviations: AKI, acute kidney injury; CK, creatine kinase.

Biochemistry revealed markedly elevated CK (79,038 U/L) and lactate dehydrogenase (LDH, 1963 U/L), confirming severe skeletal muscle injury. Serum creatinine was mildly elevated at 1.3 mg/dL, corresponding to an approximate creatinine‐based estimated glomerular filtration rate (eGFRcr) of 79 mL/min/1.73 m^2^ using the CKD‐EPI 2021 equation, while urea and electrolytes—including potassium, calcium, magnesium, and phosphorus—were within normal ranges, indicating absence of significant metabolic derangement at presentation. In the absence of a known baseline creatinine or urine‐output criteria, this isolated creatinine value was not considered sufficient to confirm AKI.

Urinalysis showed dark brown urine with 3+ dipstick positivity for blood and trace protein, without leukocytes or nitrites. In the clinical context of extreme CK elevation, these findings are suggestive of pigmenturia, most likely due to myoglobin release, although urine myoglobin was not directly measured. Overall, the laboratory findings were consistent with severe exertional rhabdomyolysis with preserved renal function, transient creatinine elevation, and no electrolyte abnormalities at presentation.

## 4. Timeline of Laboratory Parameters

**FIGURE 1:**
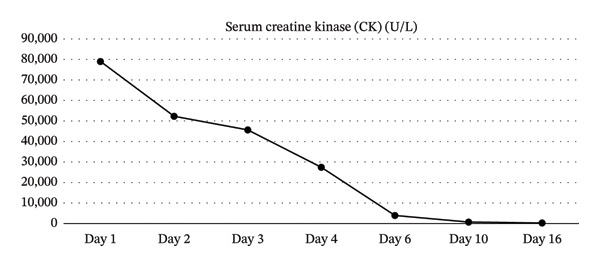
Serum CK (U/L) on a logarithmic scale from Day 1 to Day 16, illustrating decline from 79,038 U/L to 287 U/L.

**FIGURE 2:**
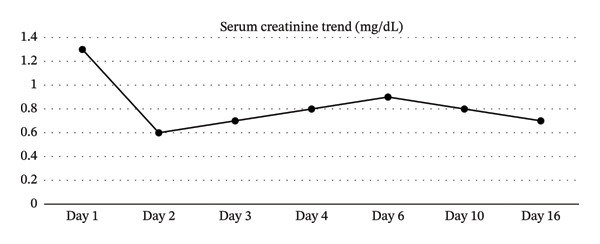
Serum creatinine (mg/dL), demonstrating a single borderline admission value with normalization by Day 2 and stability through Day 16.

**FIGURE 3:**
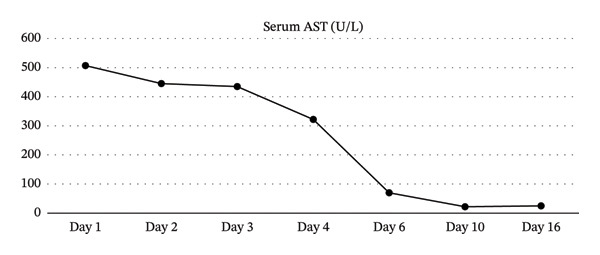
Serum AST (U/L), demonstrating decline from 507 U/L to 25 U/L by Day 16.

Serial laboratory values are presented in Table 2 and Figures 1–3. CK declined markedly from 79,038 U/L on Day 1 to 722 U/L by Day 10 and 287 U/L by Day 16 (Figure 1). Serum creatinine showed a mild initial elevation (1.3 mg/dL) with normalization within 24 h and remained stable thereafter at 0.7 mg/dL on Day 16 (Figure 2), supporting transient creatinine elevation with preserved renal function rather than confirmed AKI. AST decreased in parallel with CK, returning to normal by Day 10 and remaining normal at 25 U/L by Day 16; ALT also improved to 42 U/L by Day 16 (Figure 3). Urine dipstick blood was positive on admission and became negative by Day 2, consistent with resolution of dipstick pigment positivity.

**TABLE 2:** Serial laboratory trends (Day 1 to Day 16).

Day	CK (U/L)	Creatinine (mg/dL)	Approx. eGFRcr (mL/min/1.73 m^2^)	AST (U/L)	ALT (U/L)	Urine dipstick blood
Day 1	79,038	1.3	79	507	117	3+
Day 2	52,334	0.6	138	445	127	Negative
Day 3	45,684	0.7	132	435	153	Negative
Day 4	27,454	0.8	127	322	160	Negative
Day 6	3916	0.9	122	70	107	Negative
Day 10	722	0.8	127	22	52	Negative
Day 16	287	0.7	132	25	42	Not repeated

*Note:* ALT, alanine aminotransferase; AST, aspartate aminotransferase; eGFRcr, estimated glomerular filtration rate based on creatinine. eGFRcr values are approximate and are not intended to confirm or exclude AKI in isolation.

Abbreviation: CK, creatine kinase.

Overall, the biochemical trajectory is consistent with expected kinetics following acute muscle injury, characterized by rapid CK decline after cessation of injury, continued improvement by Day 16, effective clearance of suspected pigmenturia, and no evidence of sustained renal dysfunction.

## 5. Discussion

This case demonstrates that markedly elevated CK does not reliably predict renal injury in exertional rhabdomyolysis. Although CK reflects the extent of muscle damage, the risk of AKI depends on factors such as volume status, patient susceptibility, and timing of intervention [2, 3]. The patient was new to structured high‐intensity gym workouts, which likely increased his susceptibility to exertional muscle injury. In this patient, early fluid resuscitation likely preserved renal perfusion and facilitated myoglobin clearance, resulting in only transient creatinine elevation [2, 5].

A recently published contrasting case by Khan et al. reported exertional rhabdomyolysis with a similar CK magnitude of approximately 80,000 U/L but with significant AKI requiring renal replacement therapy [6]. Compared with that report, the present patient showed rapid creatinine normalization, stable electrolytes, no oliguria, and no need for renal replacement therapy. This contrast supports the central message that CK magnitude alone is an imperfect predictor of renal outcome in exertional rhabdomyolysis.

AKI in rhabdomyolysis is mediated by myoglobin‐induced tubular toxicity, oxidative injury, and intratubular cast formation, particularly in hypovolemic states [1, 2]. However, definite AKI could not be confirmed in this case because baseline creatinine and urine‐output criteria were unavailable. The admission creatinine of 1.3 mg/dL corresponded to an approximate eGFRcr of 79 mL/min/1.73 m^2^, and renal function normalized within 24 h. The absence of electrolyte abnormalities also supported a low‐risk clinical course with preserved renal function.

The patient weighed 70 kg, was 177‐cm tall, denied creatine monohydrate use, and reported daily hydration of more than 2.5 L. Nevertheless, transient exercise‐related fluid loss in a tropical climate may have contributed to mild prerenal creatinine elevation at presentation.

CK kinetics in this case followed expected physiological patterns, with a steady decline reflecting cessation of muscle injury. Clinically, a downward CK trend is more informative than absolute values. Elevated AST likely originated from skeletal muscle rather than liver injury [7], and ALT elevation was interpreted similarly because it improved in parallel with CK, reaching 42 U/L by Day 16. Dipstick‐positive blood without red blood cells was suggestive of myoglobinuria, although urine myoglobin was not directly measured [1, 8].

Overall, this case underscores the importance of early recognition, prompt fluid therapy, and careful renal risk interpretation. Very high CK and pigmenturia should prompt close monitoring but should not automatically be interpreted as confirmed AKI when renal function rapidly normalizes and metabolic derangements are absent.

## 6. Limitations

A key limitation of this case is the absence of baseline creatinine, urine‐output data for formal KDIGO staging, and urine myoglobin measurements, which limits definitive AKI confirmation and prevents direct biochemical confirmation of myoglobinuria. Although the admission creatinine was mildly elevated, the calculated eGFRcr was approximately 79 mL/min/1.73 m^2^ and renal function normalized within 24 h, supporting preserved renal function rather than confirmed AKI. Follow‐up laboratory data were available till Day 16 and showed continued recovery, but long‐term follow‐up beyond this point was not available. Nevertheless, the clinical and biochemical trajectory is consistent with transient creatinine elevation and a favorable prognosis.

## 7. Conclusion

This case demonstrates that severe exercise‐induced rhabdomyolysis, even with markedly elevated CK levels, can have a favorable clinical course when recognized early and managed with prompt, aggressive fluid resuscitation. Renal function trends, rather than absolute CK values, provide more meaningful insight into the risk of AKI. In this patient, renal function was preserved, and the transient creatinine elevation did not confirm definite AKI. The case also highlights key diagnostic considerations, including suspected pigmenturia and muscle‐derived transaminase elevation. As high‐intensity exercise becomes increasingly common, early clinical recognition, timely intervention, and careful renal risk interpretation remain essential to prevent complications and ensure optimal outcomes.

## Funding

No funding was received.

## Ethics Statement

Ethical approval was not required for this case report in accordance with institutional policy.

## Consent

Written informed consent was obtained from the patient.

## Conflicts of Interest

The authors declare no conflicts of interest.

## Data Availability

Data sharing is not applicable to this article as no datasets were generated or analyzed during the current study.
